# Protective role of circRNA *CCND1* in ulcerative colitis via miR-142-5p/NCOA3 axis

**DOI:** 10.1186/s12876-023-02641-6

**Published:** 2023-01-19

**Authors:** Ping Xiang, Tingrui Ge, Jingyi Zhou, Yonggang Zhang

**Affiliations:** Department of Anorectal Surgery, The First People’s Hospital of Lianyungang, No. 6 Zhenhua Road, Haizhou District, Lianyungang, 222000 China

**Keywords:** Ulcerative colitis, circRNA *CCND1*, miR-142-5p, NCOA3

## Abstract

**Background:**

Increasing research indicates that circular RNAs (circRNAs) play critical roles in the development of ulcerative colitis (UC). This study aimed to determine the role of circRNA *CCND1* in UC bio-progression, which has been shown to be downregulated in UC tissues.

**Methods:**

Reverse transcription quantitative polymerase chain reaction was used to determine the levels of circRNA *CCND1*, miR-142-5p, and nuclear receptor coactivator-3 (*NCOA3*) in UC tissues and in lipopolysaccharide (LPS)-induced Caco-2 cells. Target sites of circRNA *CCND1* and miR-142-5p were predicted using StarBase, and TargetScan to forecast potential linkage points of *NCOA3* and miR-142-5p, which were confirmed by a double luciferase reporter-gene assay. Cell Counting Kit 8 and flow cytometry assays were performed to assess Caco-2 cell viability and apoptosis. TNF-α, IL-1β, IL-6, and IL-8 were detected using Enzyme-Linked Immunosorbent Assay kits.

**Results:**

CircRNA *CCND1* was downregulated in UC clinical samples and LPS-induced Caco-2 cells. In addition, circRNA *CCND1* overexpression suppressed LPS-induced apoptosis and inflammatory responses in Caco-2 cells. Dual-luciferase reporter-gene assays showed that miR-142-5p could be linked to circRNA *CCND1*. Moreover, miR-142-5p was found to be highly expressed in UC, and its silencing inhibited LPS-stimulated Caco-2 cell apoptosis and inflammatory responses. Importantly, *NCOA3* was found downstream of miR-142-5p. Overexpression of miR-142-5p reversed the inhibitory effect of circRNA *CCND1*-plasmid on LPS-stimulated Caco-2 cells, and the effects of miR-142-5p inhibitor were reversed by si-*NCOA3*.

**Conclusion:**

CircRNA *CCND1* is involved in UC development by dampening miR-142-5p function, and may represent a novel approach for treating UC patients.

**Supplementary Information:**

The online version contains supplementary material available at 10.1186/s12876-023-02641-6.

## Introduction

As a chronic disabling inflammatory bowel disease (IBD), ulcerative colitis (UC) generally begins in young adulthood and continues throughout life [1]. The typical symptoms of UC include abdominal pain and diarrhea mixed with mucus and blood. Despite treatment efforts, the etiology and pathogenesis of UC remain unclear [2, 3]. Although diagnostic and treatment methods for UC have improved, the prognosis remains poor. In recent years, biomarker-based diagnoses and treatments have received extensive attention [4]. Thus, there is an urgent need to clearly determine the mechanisms of pathogenesis and identify effective biomarkers for UC patients.

Circular RNAs (circRNAs) are a class of non-coding RNA molecules that do not have a 5′ end cap and a 3′ terminal poly(A) tail and form a circular structure via covalent bonds. CircRNAs are formed by back splicing via non-canonical splicing [5]. CircRNAs are a type of RNA molecule without translation ability and have been confirmed to control tumor bio-functions, such as chemotherapeutic resistance, epithelial-mesenchymal transition (EMT), and cell proliferation [6–8]. A number of reports have indicated that certain circRNAs are related to the pathogenesis of UC; for instance, circRNA 0001021 regulates UC by sponging miR-224-5p [9]. IL-3 is involved in UC through circular RNA circPan3 [10]. In ulcerative colitis, circRNA Atp9b is overexpressed [11]. CircRNA *CCND1* is a new circRNA that has been shown to expedite cell metastasis and proliferation in hepatocellular carcinoma tumorigenesis by regulating the miR-497-5p/HMGA2 axis [12]. In addition, circRNA *CCND1* has been reported to be involved in laryngeal squamous cell carcinoma [13]. Although the role of circRNA *CCND1* in human cancer has been reported, the underlying mechanism of its regulation in UC is unclear.

MicroRNAs (miRNAs) are conserved small RNAs with lengths of approximately 18–20 bp [14]. Studies have found that many miRNAs are involved in the development of many diseases by post-translational regulation [15, 16]. Recently, a number of studies regarding miRNAs in UC have reported a critical role of miRNAs during the development of UC, such as miR-182-5p [17] and miR-29c-3p [18]. Additionally, miRNA-142-5p is involved in cervical cancer [19]. However, the function of miR-142-5p and its related mechanisms in UC have not yet been clarified.

NCOA3 belongs to the SRC/p160 family of nuclear receptor coactivators. NCOA3 directly binds to nuclear receptors and stimulates transcriptional activity in a hormone-dependent manner [20]. Previous research has shown that NCOA3 is associated with the biological functions of several types of human diseases, such as hepatocellular carcinoma [21] and chronic kidney disease [22]; however, a correlation between NCOA3 and UC has not been detected.

Therefore, further exploration of the pathogenesis of UC is necessary for the development of novel treatment strategies with important practical significance. In summary, this study aimed to determine whether circRNA *CCND1* regulates the UC process via the miR-142-5p/NCOA3 axis. Our study identified novel biomarkers for UC treatment.

## Methods and reagents

All methods were carried out in accordance with relevant guidelines.

### Clinical sample collection

Twenty colonic mucosa samples from patients and healthy individuals were obtained from The First People’s Hospital of Lianyungang. Study inclusion criteria were as follows: (1) none of the patients received anti-UC therapy before surgery, and (2) final diagnosis was identified by pathological determination. Exclusion criteria: (1) patients who underwent prior therapy. This study was approved by the Ethics Committee of the First People’s Hospital of Lianyungang, and each patient provided written informed consent. The tissues were stored at − 80 ° before use.

### Cell cultured

The human colorectal adenocarcinoma cell lines Caco-2 and 293T used for the dual-luciferase reporter-gene assay were obtained from American Type Culture Collection (ATCC; Manassas, VA, USA). The cells were cultured in Ham’s F-12 kmedium (Gibco, NY) supplemented with 1% penicillin and streptomycin (Thermo Fisher, USA) and 10% fetal bovine serum (Gibco, NY) in an atmosphere of 5% CO_2_ at 37 °C.

Caco-2 cells were treated with LPS (L8880, Solarbio) to establish the UC model in vitro. In brief, Caco-2 cells were incubated with 10 ng/mL LPS for 24 h, and then Caco-2 cells were harvested for subsequent experiments.

### Bioinformatic analysis

Binding sites of miR-142-5p on circRNA *CCND1* were predicted using StarBase (https://starbase.sysu.edu.cn/), and *NCOA3* fragments containing miR-142-5p binding sites were predicted using TargetScan 7.2 (https://www.targetscan.org/vert_80/).

### Dual-luciferase reporter-gene assay

Wild-type (WT) or mutant type (MUT) sequences of circRNA *CCND1* containing putative target sites for miR-142-5p and *NCOA3* were also synthesized into the pMirTarget vector (cat. no. PS100062; OriGene Technologies, Inc.) using a luciferase activity assay. Subsequently, 293T cells were co-transfected with circRNA *CCND1*-WT (or *NCOA3*-WT) or circRNA *CCND1*-MUT (or *NCOA3-MUT*) with mimic control and mimic of miR-142-5p using JETprime, according to the manufacturers instructions (Polyplus, France). Relative luciferase activity was measured using a reporter-gene system 24 h after infection (Promega). The results were normalized to those of Renilla luciferase.

### Cell transfection

To control the expression of miR-142-5p, inhibitors of miR-142-5p and inhibitor control (miR-142-5p inhibitor: 5′-UAAAGUAGGAAACACUACA-3′ and inhibitor control: 5′-CAAUACACCUUGUGUAGAACUU-3′), mimic of miR-142-5p (5′-CAUAAAGUAGAAAGCACUACU-3′), and mimic control (5′-UACUGAGAGACAUAAGUUGGUC-3′) were purchased from Genscript (Nanjing, China). To knock-down the expression of NCOA3, siRNA for *NCOA3* (NCOA3-siRNA: 5′-CTGCTTGAACATCCTTTGACTGG-3′) was used and non-specific control (control-siRNA: 5′-CACGATAAGACAATGTATTT-3′) was purchased from Thermo Fisher (Fermantas, USA). All sequences were transfected into cells that had grown to 60% confluence with JETprime (Polyplus, France). After 48 h of culture at 37 °C and 5% CO_2_, cells were collected after transfection.

### RT-qPCR assay

Following the supplier’s instructions, total RNA was recovered from cells using TRIzol® (Aladdin, Shanghai, China), and cDNA was generated after reverse transcription of RNA with the Titan One Tube RT-PCR Kit (Merck, USA). The expression levels of miRNAs were detected using TransScript® II Multiplex Probe One-Step RT-qPCR SuperMix UDG (Transgene, China), and RT-qPCR was performed using PerfectStart® Green qPCR SuperMix (Transgene, Nanjing). Relative expression levels were calculated using the 2^−ΔΔCt^ method.

### Cell counting kit-8 (CCK-8) assay

Cell proliferation was assessed using CCK-8 kits (Solarbio, Beijing, China). After transfection and LPS stimulation, Caco-2 cells were resuspended and split into 96-well plates at 5 × 10^3^ cells/well and incubated with 10 µL of CCK-8 reagent for 2–3 h at 37 °C and 5% CO_2_ in the dark. Optical density (OD) values were detected at 490 nm using an ultraviolet spectrophotometer (Bio-Rad, USA).

### Cell apoptosis assay

2 × 10^5^ LPS-stimulated Caco-2 cells were collected and cultured with 500 µL of a buffering agent containing 5 µL Annexin V-FITC and 5 µL Propidium Iodide (PI; Beyotime, Shanghai, China) at room temperature in the dark for 30 min. The cell apoptosis rate was analyzed by flow cytometry (Beckman Coulter, USA) and calculated using Kaluza analysis software (v.2.1.1.20653; Beckman Coulter, Inc.).

## Enzyme-linked immunosorbent assay (ELISA)

Supernatant from the cells was harvested and used for the detection of expression of inflammatory cytokines (TNF-α, IL-1β, IL-6, and IL-8). The ELISA kits were obtained from Beyotime Biotechnology (Shanghai, China). All operations were performed according to the supplier’s instructions.

### Western blot assay

To collect total protein from cells, radioimmunoprecipitation assay (RIPA) buffer (Beyotime Institute of Biotechnology, China) was used. The proteins were loaded in 10% SDS-PAGE gel and then transferred onto polyvinylidene fluoride (PVDF) membranes. After blocking with 5% skim milk in PBS-Tween 20 (PBST) solution at room temperature for 1 h, the membranes were incubated with primary antibodies (NCOA3, 1:1000, ab133611, abcam, Cambridge, MA, USA; GAPDH, 1:10000, EA015, ELK Biotechnology, Wuhan, China) overnight at 4 ℃. The membranes were subsequently incubated with the secondary antibody at room temperature for 2 h. Finally, to visualize protein bands, an enhanced chemiluminescence (ECL) substrate (Cytiva, USA) was performed according to the manufacturer’s protocol. The original blots were presented in the additional file 1. It should be noted that during the western blot assay, we first cut out the corresponding membrane according to the molecular weight of the target protein prior to hybridisation with antibody, and then the corresponding membrane was incubated the primary antibody. Therefore the original blots is not a full length membranes.

### Statistical analysis

The mean ± standard deviation (SD) represents the data from triplicate experiments. Statistical comparisons among groups were performed using Student’s *t*-test or one-way ANOVA followed by Tukey’s post hoc test. Statistical significance was set at P < 0.05.

## Results

### CircRNA *CCND1* is down-regulated in UC

**Fig. 1:**
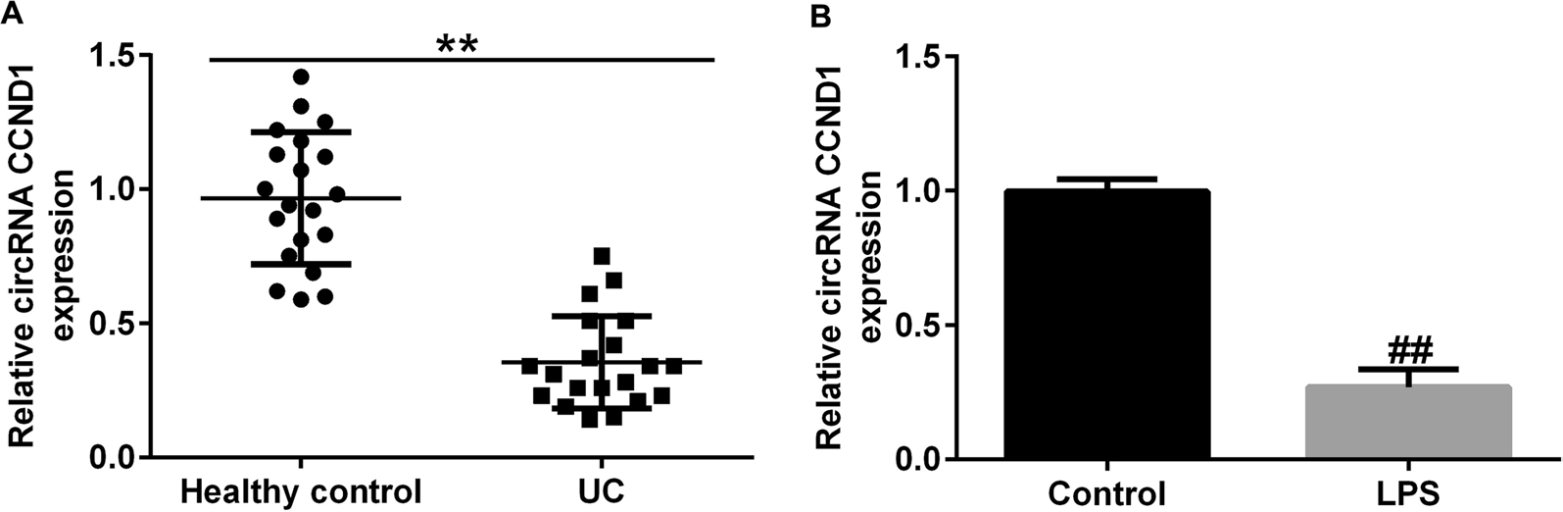
CircRNA *CCND1* levels were diminished in UC. **A** CircRNA *CCND1* levels in colonic mucosa samples and normalized to control. **B** CircRNA *CCND1* levels in LPS-treated Caco-2 and control cells. Data are shown as means ± SD of three replicate experiments. **p < 0.01 versus healthy control; ^##^p < 0.01 versus control

Owing to the significant function of circRNA *CCND1* in different diseases, this study explored the effect of circRNA *CCND1* on UC. We performed RT-qPCR analysis of circRNA *CCND1* expression levels in UC. The results revealed that circRNA *CCND1* was at a low level in UC samples in contrast to normal tissues (Fig. 1A). CircRNA *CCND1* expression was down-regulated in 10 ng/mL LPS-treated Caco-2 cells (Fig. 1B). Collectively, circRNA *CCND1* is down-regulated in UC, thus it is involved in the UC process.

### MiR-142-5p binds circRNA *CCND1*

**Fig. 2:**
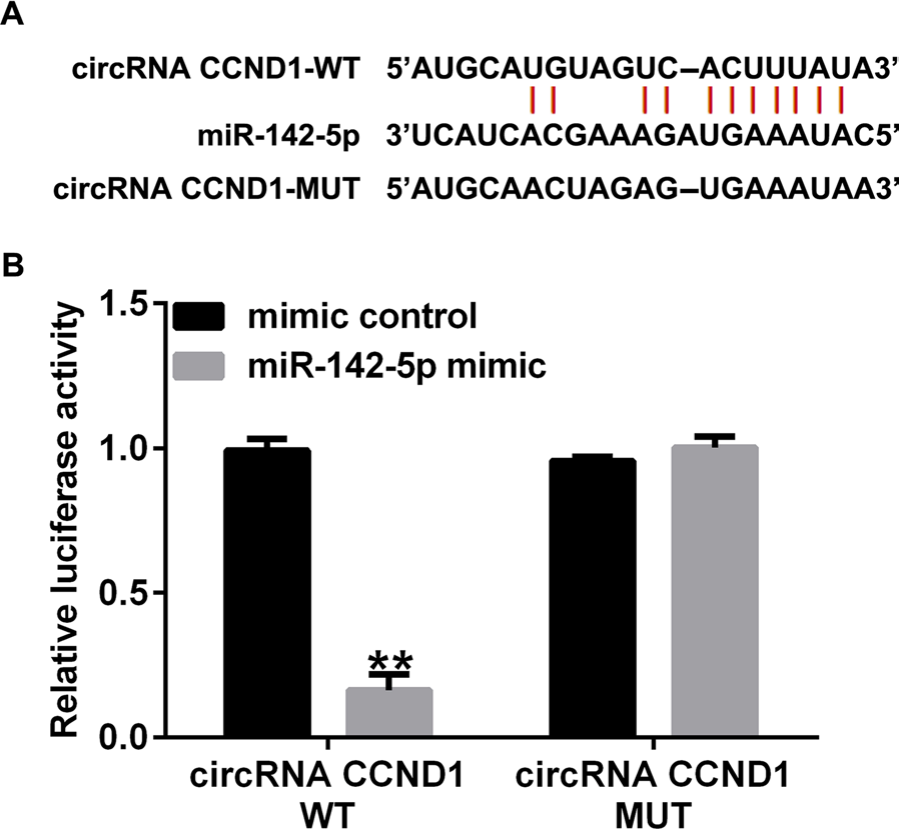
CircRNA *CCND1* targets miRNA-142-5p. **A** The conjunction points of miRNA-142-5p on circRNA *CCND1*. **B** Luciferase reporter-gene activity of miR-142-5p co-transfection with circRNA *CCND1* WT and MUT. Data are shown as means ± SD of three replicate experiments. **p < 0.01 versus mimic control

To investigate the potential binding sites of circRNA *CCND1* and miR-142-5p, bioinformatic prediction tools (StarBase) were used, and we discovered that miR-142-5p possibly contained circRNA *CCND1* binding sites (Fig. 2A). To confirm the link between circRNA *CCND1* and miR-142-5p, a dual-luciferase reporter-gene assay was performed using 293T cells. As shown in Fig. 2B, the relative luciferase level of circRNA *CCND1* 3ʹ-UTR was notably reduced when cells were co-cultured with a mimic of miR-142-5p. When potential binding sites were mutated, the miR-142-5p mimic exhibited no effect. These results confirmed that miRNA-142-5p is sponged by circRNA *CCND1*.

### MiR-142-5p is up-regulated in UC

**Fig. 3:**
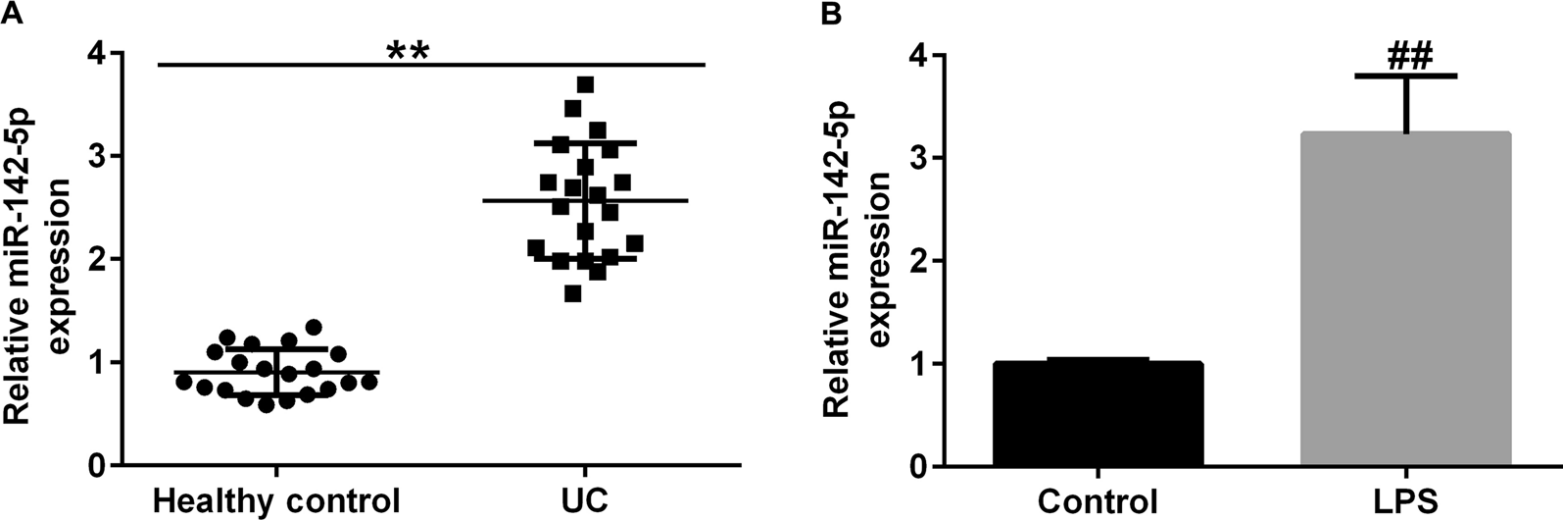
MiR-142-5p levels elevated in UC. **A** miR-142-5p levels in colonic mucosa samples and normalized to control. **B** miR-142-5p level in LPS-treated Caco-2 and control cells. Data are shown as means ± SD of three replicate experiments. **p < 0.01 versus Healthy control; ^##^p < 0.01 versus control

The expression level of miR-142-5p was measured by RT-qPCR and found to be upregulated in UC tissues (Fig. 3A). Consistently, miR-142-5p was highly expressed in LPS-induced Caco-2 cells (Fig. 3B).

### Overexpression of circRNA *CCND1* disturbs miR-142-5p in Caco-2 cells

**Fig. 4:**
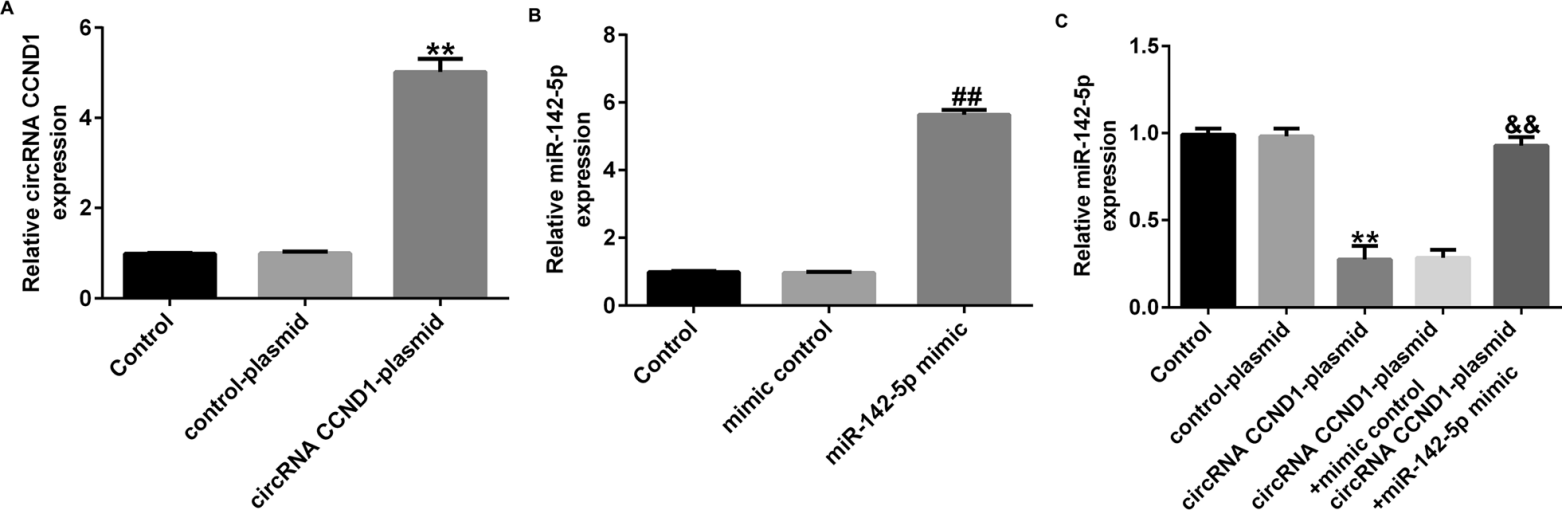
miR-142-5p was negatively regulated by circRNA *CCND1*. **A** Efficiency of circRNA *CCND1*-plasmid transfection. **B** Efficiency of miR-142-5p mimic transfection. **C** Levels of miR-142-5p with circRNA *CCND1*-plasmid and miR-142-5p mimic co-transfection as determined by RT-qPCR. Data are shown as means ± SD of three replicate experiments. **p < 0.01 versus control-plasmid; ^##^p < 0.01 versus mimic control; ^&&^p < 0.01 versus circRNA *CCND1*-plasmid + mimic control

To investigate the relationship between circRNA *CCND1* and miR-142-5p, RT-qPCR was performed to measure circRNA *CCND1* and miR-142-5p expression levels. Compared to the control plasmid group, circRNA *CCND1* was up-regulated in Caco-2 cells transfected with circRNA *CCND1*-plasmid (Fig. 4A). miR-142-5p was upregulated when miR-142-5p mimic was transfected, in contrast to that in the mimic control group (Fig. 4B). RT-qPCR results revealed that miR-142-5p expression was inhibited in Caco-2 cells transfected with circRNA *CCND1*-plasmid, while co-transfection with a mimic of miR-142-5p reduced its levels (Fig. 4C). These results indicated that circRNA *CCND1* regulates miR-142-5p expression in Caco-2 cells.

### Overexpression of circRNA *CCND1* repressed cell apoptosis and inflammatory responses via miR-142-5p in LPS-treated Caco-2 cells

**Fig. 5:**
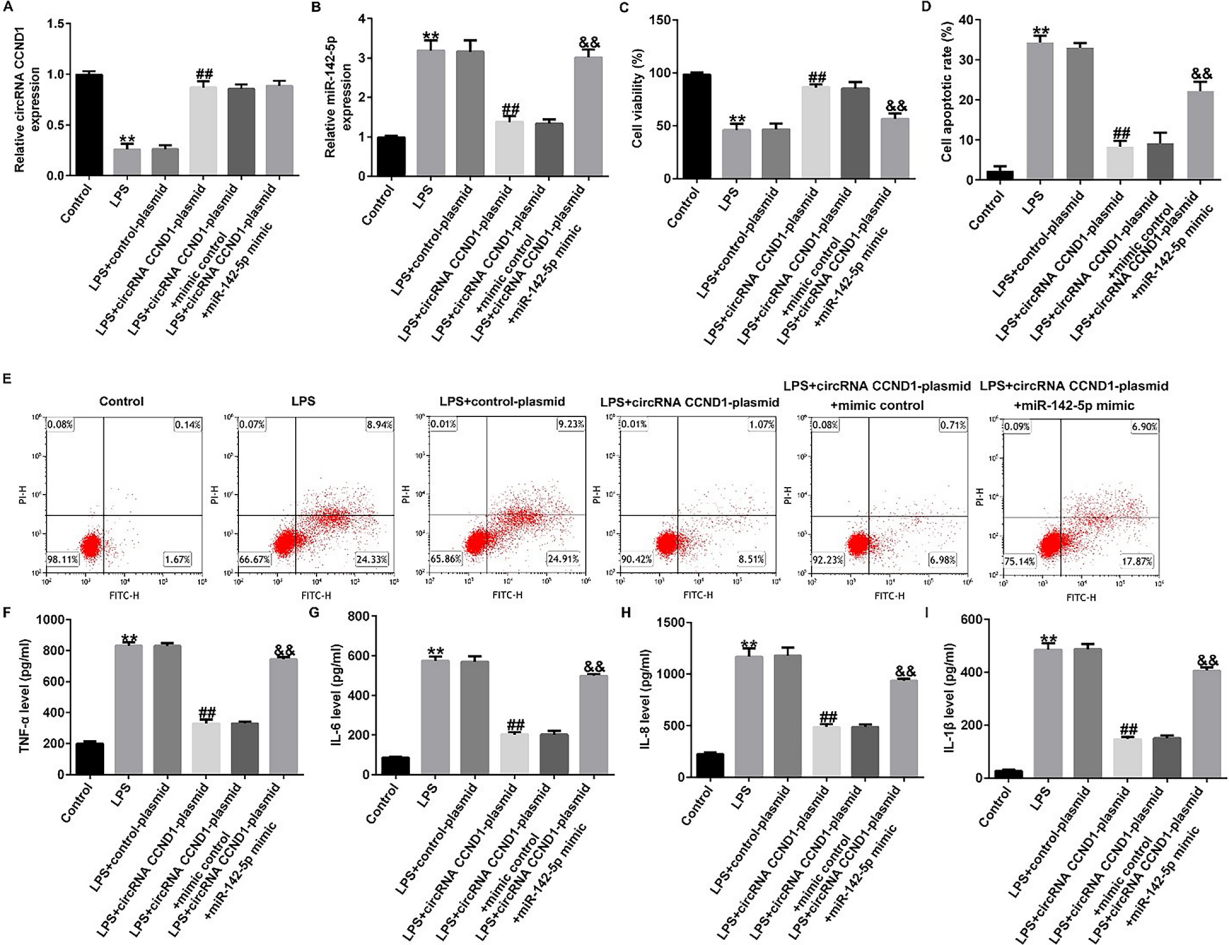
CircRNA ***CCND1*** inhibits LPS-induced Caco-2 cell injury by downregulating miR-142-5p expression. **A**, **B** RT-qPCR uncovered the levels of miRNA-142-5p and circRNA *CCND1* in different cells. **C** Cell viability was determined by CCK-8 assays. **D**, **E** Apoptosis ratio of LPS-induced cells was detected by flow cytometry. **F**–**I** Inflammatory cytokine levels were measured by ELISA. Data are shown as means ± SD of three replicate experiments. **p < 0.01 versus Control; ^##^p < 0.01 versus LPS + control-plasmid; ^&&^p < 0.01 versus LPS + circRNA *CCND1*-plasmid + mimic control

To investigate whether circRNA CCND1 functions by inhibiting miR-142-5p expression, Caco-2 cells were co-transfected with circRNA *CCND1*-plasmid and miR-142-5p mimic. RT-qPCR analysis confirmed that circRNA *CCND1* was downregulated in LPS-induced cells and miR-142-5p was significantly upregulated, compared with the control group. In the LPS plus circRNA *CCND1*-plasmid group, the circRNA *CCND1*-plasmid enhanced its expression level and knocked-down miR-142-5p levels in contrast with the LPS + control-plasmid group, and miR-142-5p expression levels were restored when the miR-142-5p mimic was co-transfected (Fig. 5A, B). CCK-8 results indicated that the viability of LPS-treated Caco-2 cells was lower than that of the control; however, overexpression of circRNA *CCND1* rescued the inhibitory effect of LPS treatment, while co-transfection of the miR-142-5p mimic diminished the phenomenon (Fig. 5C). The flow cytometry assay revealed that LPS led to cell apoptosis, the circRNA *CCND1*-plasmid transfected into LPS-treated Caco-2 cells decreased apoptosis, and co-transfection of miR-142-5p mimic increased the percentage of apoptosis (Fig. 5D, E). The concentrations of TNF-α, IL-6, IL-8, and IL-1β were all strongly enhanced by LPS and impaired by circRNA *CCND1*-plasmid transfection, which was reversed by the miR-142-5p mimic (Fig. 5F–I).

### NCOA3 acts downstream of miR-142-5p

**Fig. 6:**
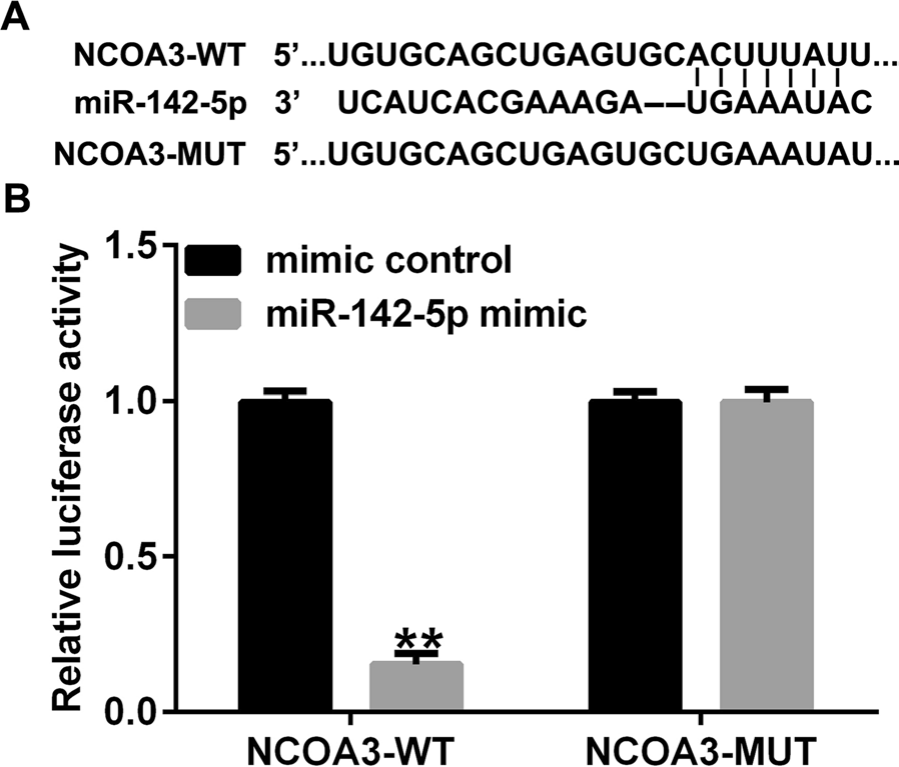
***NCOA3*** is a downstream mRNA of miRNA-142-5p. **A** The predicted binding sites between miRNA-142-5p and *NCOA3* were predicted using TargetScan 7.0. **B** A dual relative luciferase assay was used to confirm the linkage between miRNA-142-5p and *NCOA3*. Data are shown as means ± SD of three replicate experiments. **p < 0.01 versus mimic control

We then tested the downstream mRNA of miR-142-5p using Targetscan 7.0, and found that *NCOA3* contained potential miR-142-5p binding sites (Fig. 6A). We used the dual-relative luciferase method to address this binding. We demonstrated that overexpression of miR-142-5p downregulated the luciferase level of the *NCOA3*-WT reporter gene (Fig. 6B).

### MiRNA-142-5p modulates NCOA3 expression in UC cells

**Fig. 7:**
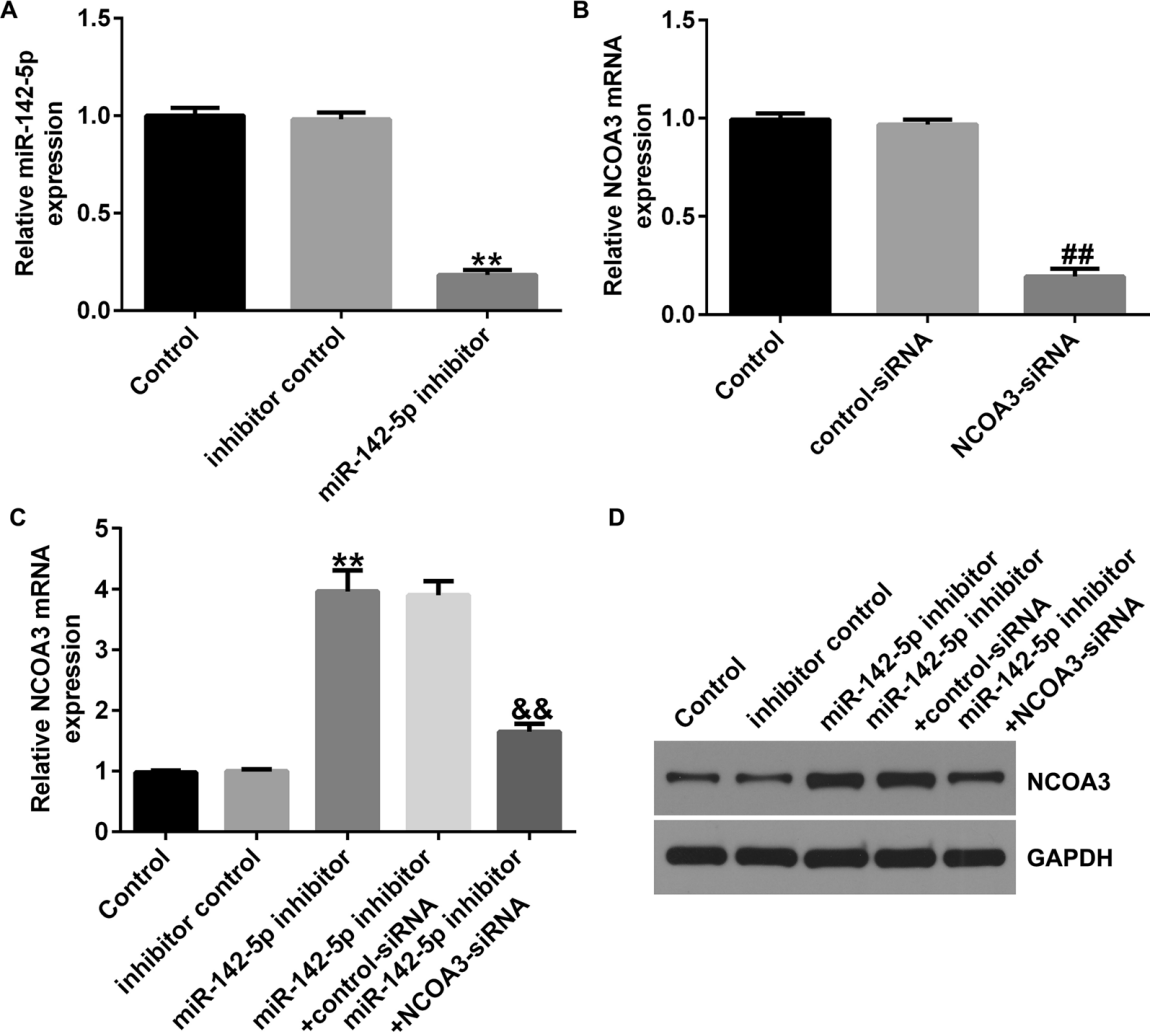
NCOA3 was negatively controlled by miR-142-5p. **A** Efficiency of miR-142-5p inhibitor transfection. **B** Efficiency of *NCOA3*-siRNA transfection. **C**, **D** Levels of *NCOA3* with miR-142-5p inhibitor and *NCOA3*-siRNA co-transfection as determined by RT-qPCR and western blot analysis. Data are shown as means ± SD of three replicate experiments. **p < 0.01 versus inhibitor control; ^##^p < 0.01 versus control-siRNA; ^&&^p < 0.01 versus miR-142-5p inhibitor + control-siRNA.

To understand the relationship between NCOA3 and miR-142-5p, RT-qPCR was used to count *NCOA3* and miR-142-5p expression levels. miR-142-5p expression was downregulated by miR-142-5p inhibitor transfection compared to the inhibitor control group (Fig. 7A). *NCOA3* was downregulated when *NCOA3*-siRNA was transfected, in contrast to that in the control siRNA group (Fig. 7B). Furthermore, we found that the inhibition of miR-142-5p increased the mRNA and protein levels of NCOA3 in Caco-2 cells, but *NCOA3*-siRNA co-transfection reversed this outcome (Fig. 7C, D). These results indicated that *NCOA3* is a target of miR-142-5p and that the level of NCOA3 is negatively associated with miR-142-5p.

### NCOA3 participated in impact of mir-142-5p silencing in LPS-induced Caco-2 cells

**Fig. 8:**
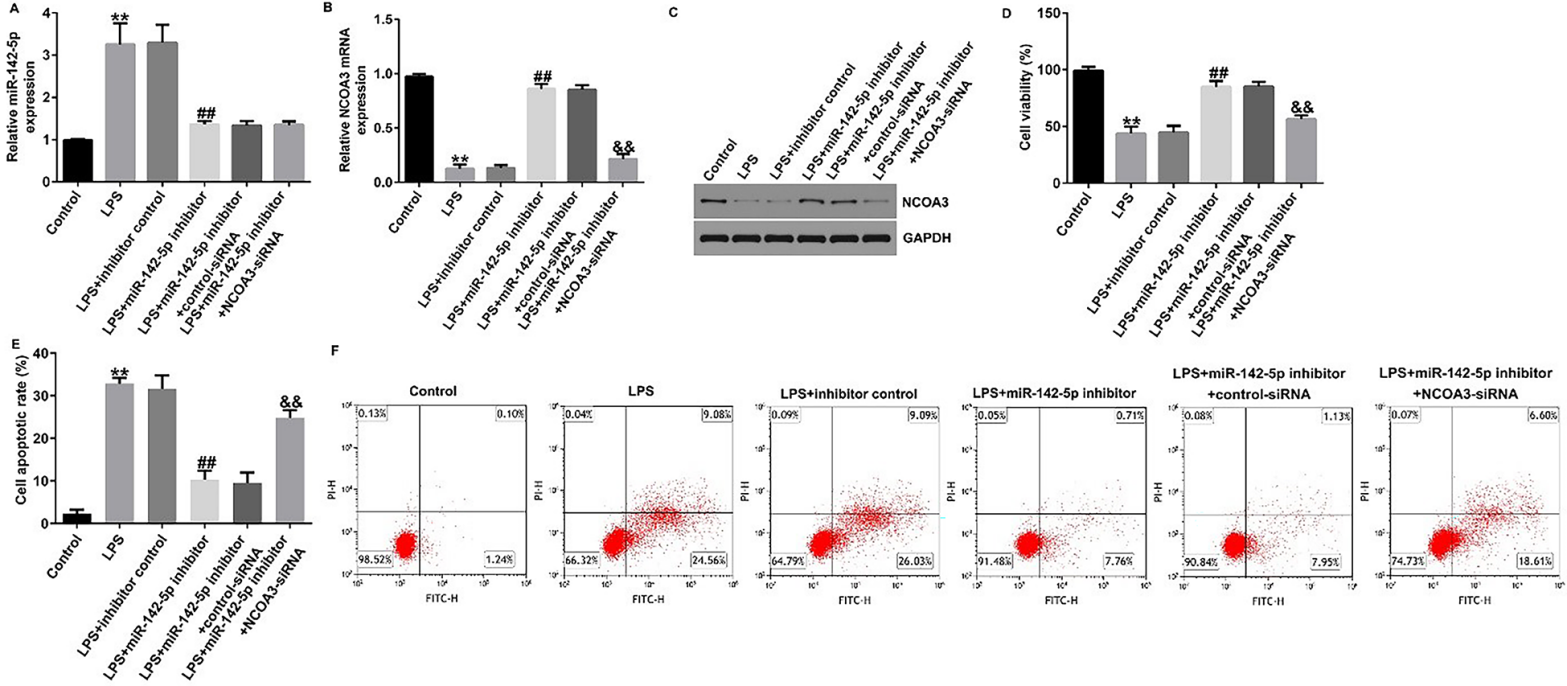
Inhibition of miR-142-5p suppressed LPS-induced cell apoptosis in Caco-2 cells through *NCOA3*. **A**, **B** RT-qPCR revealed the levels of miRNA-142-5p and *NCOA3* in different cells. **C** Western blot assay to analyze NCOA3 protein levels in different cells. **D** Cell viability was counted using CCK-8 kits. **E**, **F** Apoptosis ratio of LPS-induced cells was detected by flow cytometry. Data are shown as means ± SD of three replicate experiments. **p < 0.01 versus Control; ^##^p < 0.01 versus LPS + inhibitor control; ^&&^p < 0.01 versus LPS + miR-142-5p inhibitor + control-siRNA

**Fig. 9:**
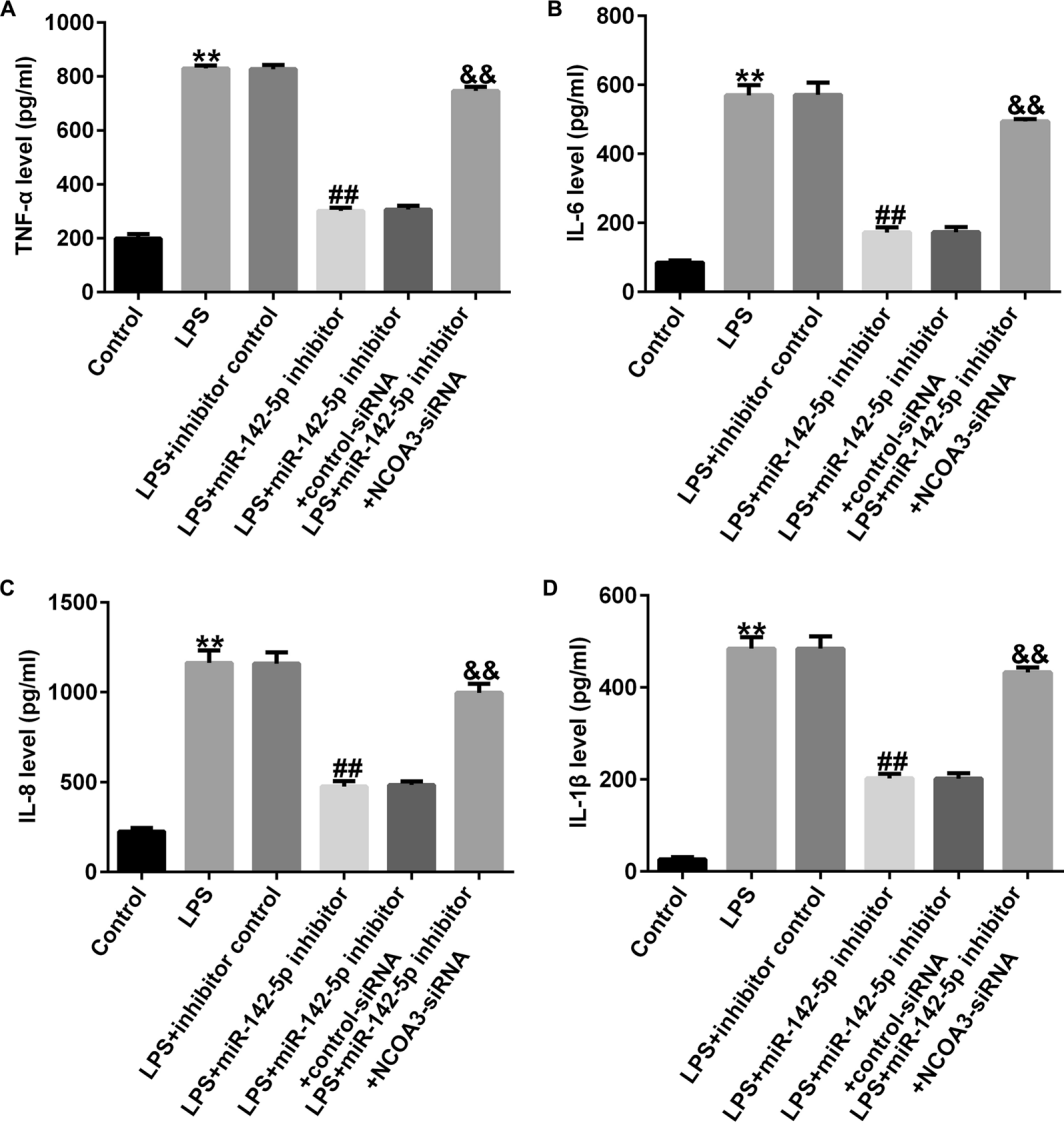
Inhibition of miR-142-5p suppressed LPS-induced cell inflammation in Caco-2 cells through *NCOA3*. **A**–**D** Inflammatory cytokines were measured by ELISA. Data are shown as means ± SD of three replicate experiments. **p < 0.01 versus Control; ^##^p < 0.01 versus LPS + inhibitor control; ^&&^p < 0.01 versus LPS + miR-142-5p inhibitor + control-siRNA

To determine whether miR-142-5p functions by inhibiting NCOA3 expression, Caco-2 cells were co-infected with a miR-142-5p inhibitor and *NCOA3*-siRNA. RT-qPCR and western blot analysis indicated that miR-142-5p was highly expressed in the LPS-treated model and NCOA3 was down-regulated compared with the control group. In the LPS + miR-142-5p inhibitor group, miR-142-5p inhibitor abolished its expression and restored NCOA3 levels in contrast with LPS + inhibitor control, whereas NCOA3 expression was absent when *NCOA3*-siRNA was co-transfected (Fig. 8A–C). CCK-8 results showed that the viability of LPS-induced Caco-2 cells was lower than that of the control group; however, inhibition of miR-142-5p restored the viability of LPS-stimulated Caco-2 cells, whereas co-transfection of *NCOA3*-siRNA reversed this effect (Fig. 8D). FCS revealed that LPS exposure accelerated the cell apoptotic rate, and miR-142-5p inhibitor transfection of LPS-treated Caco-2 cells could reduce apoptosis, whereas co-transfection with *NCOA3*-siRNA increased the percentage of apoptotic cells (Fig. 8E, F). The concentrations of TNF-α, IL-6, IL-8, and IL-1β were all notably elevated by LPS treatment and diminished by the miR-142-5p inhibitor, which was reversed by NCOA3 downregulation (Fig. 9A–D).

## Discussion

UC is a chronic non-specific IBD characterized by chronic inflammation and ulcerative changes to the intestinal mucosa. UC lesions are primarily located in the mucosa, colonic submucosa, and rectum. However, its pathogenesis remains unclear. Some studies suggest that dysfunction of the immune system is an important factor in UC-induced intestinal inflammation and tissue damage, and others suggest that it may be related to environmental, genetic, infectious, and immune factors. The clinical treatment of UC is usually based on the use of corticosteroids, immunosuppressants, and aminosalicylates [23–25].

In this study, we elaborated on the molecular mechanism by which circRNA *CCND1* relieved UC induced by LPS-stimulation. This examination confirmed that circRNA *CCND1* relieved UC progression via miR-142-5p, indicating that circRNA *CCND1* may serve as a novel biomarker for UC.

Accumulating evidence has shown that circRNAs play a critical role in UC. In UC, circRNAs have been described as biomarkers. circRNA-*SOD2* is involved in UC [26]. Xu et al. found that circRNA-*HECTD1* promotes autophagy and affects UC [27]. In contrast, circRNA-102,610 is upregulated in UC and promotes EMT via miR-130a-3p [28]. However, few studies have investigated the role of the circRNA *CCND1* in UC. In our study, we found that circRNA *CCND1* was disturbed in UC. These findings increase the recent understanding of the role of circRNA *CCND1* in the modulation of UC, which may be helpful in better understanding the pathological mechanisms underpinning UC.

Many researchers have illustrated that circRNAs regulate disease processes via miRNAs [29, 30]. Zhou found that many miRNAs are involved in UC [31]. Li Z indicated that miR-146a and miR-196 are associated with UC [32]. Wu et al. showed that miR-223-3p ameliorates UC via pyroptosis [33]. In contrast, miR-21 and miR-155 repress UC [34]. Here, experimental evidence identified miRNA-142-5p as a downstream target of circRNA *CCND1*, with confirmed binding sites. We found that the level of miRNA-142-5p was clearly upregulated and was controlled by the level of circRNA *CCND1* in UC. Moreover, the data of current study indicated that overexpression of circRNA *CCND1* repressed cell apoptosis and inflammatory responses in LPS-treated Caco-2 cells through down-regulating the expression of miRNA-142-5p.

Previously, NCOA3 was reported to play a critical role in human diseases such as breast cancer [35], osteoarthritis [36], hepatic injury, and fibrosis [37]. In this study, we confirmed that NCOA3 was a direct target of miR-142-5p, and it was negatively regulated by miR-142-5p in Caco-2 cells. In addition, the findings revealed that miR-142-5p silencing relieved LPS-induced Caco-2 cells injury through targeting NCOA3, suggesting the important role of NCOA3 in UC development.

This study is the first to clarify the expression of circRNA *CCND1* in UC, and clarify the possible molecular mechanism of its involvement in the occurrence and development of UC. It provides more theoretical basis for the pathogenesis of UC, and provides potential targets for clinical treatment of UC. However, our study still has some limitations. For example, this study was mainly conducted on UC cell model, and no in vivo experimental study was conducted. In addition, circRNA *CCND1* may also play a role in UC by regulating other signal pathways, so it is also necessary to explore the signal pathways regulated by circRNA CCND1 in UC. We will conduct in-depth research on these issues in the next step of research.

In conclusion, our study demonstrated that circRNA *CCND1* modulates miRNA-142-5p/*NCOA3* to repress the UC process. Our data demonstrate that circRNA *CCND1*/miRNA-142-5p/*NCOA3* provides a new therapeutic strategy for UC patients.

## Supplementary Information

**Additional file 1.** The original blots.

## Data Availability

The datasets used and/or analyzed during the current study are available from the corresponding author on reasonable request.

## References

[1] Ungaro R, Mehandru S, Allen PB, et al. Ulcerative colitis. Lancet. 2017;389:1756–70. 27914657 10.1016/S0140-6736(16)32126-2 PMC6487890

[2] Chu H, Khosravi A, Kusumawardhani LP, et al. Gene–microbiota interactions contribute to the pathogenesis of inflammatory bowel disease. Science. 2016;352:1116–20. 27230380 10.1126/science.aad9948 PMC4996125

[3] Ming L, Bing W, Xiaotong S, et al. Upregulation of intestinal barrier function in rat with DSS-induced colitis by a defined bacterial consortium is associated with expansion of IL-17A producing gamma delta T cells. Front Immunol. 2017;8:824. 10.3389/fimmu.2017.00824 PMC5506203 28747917

[4] Pope JL, Bhat AA, Sharma A, et al. Claudin-1 regulates intestinal epithelial homeostasis through the modulation of Notch. Gut. 2014;63(4):622–34. 23766441 10.1136/gutjnl-2012-304241 PMC4083824

[5] Chen L, Wang C, Sun H, Wang J, Liang Y, Wang Y, Wong G. The bioinformatics toolbox for circRNA discovery and analysis. Brief Bioinform. 2021;22(2):1706–28. 10.1093/bib/bbaa001. 32103237 10.1093/bib/bbaa001 PMC7986655

[6] Xu J, Ji L, Liang Y, Wan Z, Zheng W, Song X, Gorshkov K, Sun Q, Lin H, Zheng X, Chen J, Jin RA, Liang X, Cai X. CircRNA-SORE mediates sorafenib resistance in hepatocellular carcinoma by stabilizing YBX1. Signal Transduct Target Ther. 2020;5(1):298. 10.1038/s41392-020-00375-5. 33361760 10.1038/s41392-020-00375-5 PMC7762756

[7] Liu J, Xue N, Guo Y, Niu K, Gao L, Zhang S, Gu H, Wang X, Zhao D, Fan R. CircRNA_100367 regulated the radiation sensitivity of esophageal squamous cell carcinomas through miR-217/Wnt3 pathway. Aging. 2019;11(24):12412–27. 31851619 10.18632/aging.102580 PMC6949088

[8] Huang G, Liang M, Liu H, Huang J, Li P, Wang C, Zhang Y, Lin Y, Jiang X. CircRNA hsa_circRNA_104348 promotes hepatocellular carcinoma progression through modulating miR-187-3p/RTKN2 axis and activating Wnt/β-catenin pathway. Cell Death Dis. 2020;11(12):1065. 33311442 10.1038/s41419-020-03276-1 PMC7734058

[9] Li B, Li Y, Li L, Yu Y, Gu X, Liu C, Long X, Yu Y, Zuo X. Hsa_circ_0001021 regulates intestinal epithelial barrier function via sponging mir-224-5p in ulcerative colitis. Epigenomics. 2021;13(17):1385–401. 34528447 10.2217/epi-2021-0230

[10] Zhu P, Zhu X, Wu J, He L, Lu T, Wang Y, Liu B, Ye B, Sun L, Fan D, Wang J, Yang L, Qin X, Du Y, Li C, He L, Ren W, Wu X, Tian Y, Fan Z. IL-13 secreted by ILC2s promotes the self-renewal of intestinal stem cells through circular RNA circPan3. Nat Immunol. 2019;20(2):183–94. 30643264 10.1038/s41590-018-0297-6

[11] Li F, Fu J, Fan L, Lu S, Zhang H, Wang X, Liu Z. Overexpression of circAtp9b in ulcerative colitis is induced by lipopolysaccharides and upregulates PTEN to promote the apoptosis of colonic epithelial cells. Exp Ther Med. 2021;22(6):1404. 34675997 10.3892/etm.2021.10840 PMC8524737

[12] Zheng S, Hou J, Chang Y, Zhao D, Yang H, Yang J. CircRNA Circ-CCND1 aggravates hepatocellular carcinoma tumorigenesis by regulating the miR-497-5p/HMGA2 Axis. Mol Biotechnol. 2022;64(2):178–86. 34564768 10.1007/s12033-021-00391-y

[13] Zang Y, Li J, Wan B, Tai Y. circRNA circ-CCND1 promotes the proliferation of laryngeal squamous cell carcinoma through elevating CCND1 expression via interacting with HuR and miR-646. J Cell Mol Med. 2020;24(4):2423–33. 31951319 10.1111/jcmm.14925 PMC7028846

[14] Shi Y, Circular. RNA LPAR3 sponges microRNA-198 to facilitate esophageal cancer migration, invasion, and metastasis. Cancer Sci. 2020;111(8):2824–36. 32495982 10.1111/cas.14511 PMC7419039

[15] Bandiera S. miR-122 a key factor and therapeutic target in liver disease. J Hepatol. 2015;62(2):448–57. 25308172 10.1016/j.jhep.2014.10.004

[16] Elton TS. Regulation of the MIR155 host gene in physiological and pathological processes. Gene. 2013;532(1):1–12. 23246696 10.1016/j.gene.2012.12.009

[17] Tang S, Guo W, Kang L, Liang J. MiRNA-182-5p aggravates experimental ulcerative colitis via sponging Claudin-2. J Mol Histol. 2021;52(6):1215–24. 10.1007/s10735-021-10021-1. 34623552 10.1007/s10735-021-10021-1 PMC8616881

[18] Guo J, Zhang R, Zhao Y, Wang J. MiRNA-29c-3p promotes intestinal inflammation via targeting leukemia inhibitory factor in ulcerative colitis. J Inflamm Res. 2021;14:2031–43. 34040415 10.2147/JIR.S302832 PMC8140949

[19] Ke L, Chen Y, Li Y, Chen Z, He Y, Liu J, Zhuang Y. Mir-142-5p promotes cervical cancer progression by targeting LMX1A through Wnt/β-catenin pathway. Open Med (Wars). 2021;16(1):224–36. 33585699 10.1515/med-2021-0218 PMC7862994

[20] Salazar-Silva R, Dantas VLG, Alves LU, Batissoco AC, Oiticica J, Lawrence EA, Kawafi A, Yang Y, Nicastro FS, Novaes BC, Hammond C, Kague E, Mingroni-Netto RC. NCOA3 identified as a new candidate to explain autosomal dominant progressive hearing loss. Hum Mol Genet. 2021;29(22):3691–705. 33326993 10.1093/hmg/ddaa240 PMC7823111

[21] Li W, Yan Y, Zheng Z, Zhu Q, Long Q, Sui S, Luo M, Chen M, Li Y, Hua Y, Deng W, Lai R, Li L. Targeting the NCOA3-SP1-TERT axis for tumor growth in hepatocellular carcinoma. 2020; 11(11):1011. 10.1038/s41419-020-03218-x PMC7689448 33239622

[22] Zhou T, Lin W, Lin S, Zhong Z, Luo Y, Lin Z, Xie W, Shen W, Hong K. Association of nuclear receptor coactivators with hypoxia-inducible factor-1α in the serum of patients with chronic kidney disease. Biomed Res Int, 2020; 2020:1587915. 10.1155/2020/1587915 PMC7455818 32884936

[23] Lord JD. Promises and paradoxes of regulatory T cells in inflammatory bowel disease. World J Gastroenterol. 2015;21(40):11236–45. 26523099 10.3748/wjg.v21.i40.11236 PMC4616201

[24] Liu Y, Zhang XJ, Yang CH. Oxymatrine protects rat brains against permanent focal ischemia and downregulates NF-kappa B expression. Brain Res. 2009;1268:174–80. 19285049 10.1016/j.brainres.2009.02.069

[25] Hong Li S, Lei L, Lei S. Cardioprotective effects and underlying mechanisms of oxymatrine against ischemic myocardial injuries of rats. Phytother Res. 2008;22(7):985–9. 18389484 10.1002/ptr.2452

[26] Wang TT, Han Y, Gao FF, Ye L, Zhang YJ. Effects of circular RNA circ-SOD2 on intestinal epithelial barrier and ulcerative colitis. Beijing Da Xue Xue Bao Yi Xue Ban. 2019;51(5):805–12. 31624381 10.19723/j.issn.1671-167X.2019.05.003 PMC7433506

[27] Xu Y, Tian Y, Li F, Wang Y, Yang J, Gong H, Wan X, Ouyang M. Circular RNA HECTD1 mitigates ulcerative colitis by promoting enterocyte autophagy via miR-182-5p/HuR axis. Inflamm Bowel Dis. 2022;28(2):273–88. 34427642 10.1093/ibd/izab188

[28] Yin J, Ye YL, Hu T, Xu LJ, Zhang LP, Ji RN, Li P, Chen Q, Zhu JY, Pang Z. Hsa_circRNA_102610 upregulation in Crohn’s disease promotes transforming growth factor-β1-induced epithelial-mesenchymal transition via sponging of hsa-miR-130a-3p. World J Gastroenterol. 2020;26(22):3034–55. 32587447 10.3748/wjg.v26.i22.3034 PMC7304108

[29] Zhang M, Bai X, Zeng X, Liu J, Liu F, Zhang Z. circRNA-miRNA-mRNA in breast cancer. Clin Chim Acta. 2021;523:120–30. 34537217 10.1016/j.cca.2021.09.013

[30] Chang X, Zhu G, Cai Z, Wang Y, Lian R, Tang X, Ma C, Fu S. miRNA, lncRNA and circRNA: targeted molecules full of therapeutic prospects in the development of diabetic retinopathy. Front Endocrinol (Lausanne). 2021;12:771552. 34858342 10.3389/fendo.2021.771552 PMC8631471

[31] Zhou J, Liu J, Gao Y, Shen L, Li S, Chen S. miRNA-Based potential biomarkers and new molecular insights in ulcerative colitis. Front Pharmacol. 2021;12:707776. 10.3389/fphar.2021.707776. 34305614 10.3389/fphar.2021.707776 PMC8298863

[32] Li Z, Wang Y, Zhu Y. Association of miRNA-146a rs2910164 and miRNA-196 rs11614913 polymorphisms in patients with ulcerative colitis: a meta-analysis and review. Medicine (Baltimore). 2018;97(39):e12294. 10.1097/MD.0000000000012294 PMC6181578 30278502

[33] Wu X, Pan S, Luo W, Shen Z, Meng X, Xiao M, Tan B, Nie K, Tong T, Wang X. Roseburia intestinalisderived flagellin ameliorates colitis by targeting miR2233pmediated activation of NLRP3 inflammasome and pyroptosis. Mol Med Rep. 2020;22(4):2695–704. 32700754 10.3892/mmr.2020.11351 PMC7453595

[34] Qu S, Shen Y, Wang M, Wang X, Yang Y. Suppression of miR-21 and miR-155 of macrophage by cinnamaldehyde ameliorates ulcerative colitis. Int Immunopharmacol. 2019;67:22–34. 30530166 10.1016/j.intimp.2018.11.045

[35] Burwinkel B, Wirtenberger M, Klaes R, Schmutzler RK, Grzybowska E, Försti A, Frank B, Bermejo JL, Bugert P, Wappenschmidt B, Butkiewicz D, Pamula J, Pekala W, Zientek H, Mielzynska D, Siwinska E, Bartram CR, Hemminki K. Association of NCOA3 polymorphisms with breast cancer risk. Clin Cancer Res. 2005;11(6):2169–74. 15788663 10.1158/1078-0432.CCR-04-1621

[36] Gee F, Rushton MD, Loughlin J, Reynard LN. Correlation of the osteoarthritis susceptibility variants that map to chromosome 20q13 with an expression quantitative trait locus operating on NCOA3 and with functional variation at the polymorphism rs116855380. Arthritis Rheumatol. 2015;67(11):2923–32. 26211391 10.1002/art.39278 PMC4832313

[37] Ma X, Xu L, Wang S, Chen H, Xu J, Li X, Ning G. Loss of steroid receptor co-activator-3 attenuates carbon tetrachloride-induced murine hepatic injury and fibrosis. Lab Investig. 2009;89(8):903–14. 19488034 10.1038/labinvest.2009.51 PMC3620314

