# Correction: Protective role of circrna *CCND1* in ulcerative colitis via miR-142-5p/NCOA3 axis

**DOI:** 10.1186/s12876-025-04360-6

**Published:** 2025-12-16

**Authors:** Ping Xiang, Tingrui Ge, Jingyi Zhou, Yonggang Zhang

**Affiliations:** Department of Anorectal Surgery, The First People’s Hospital of Lianyungang, No. 6 Zhenhua Road, Haizhou District, Lianyungang, 222000, China

**Correction: BMC Gastroenterol 23, 18 (2023)**

**https://doi.org/10.1186/s12876-023-02641-6**

Following publication of the original article it was reported that there were errors in Figs. 5E and 8E.

The original and corrected Figs. 5 and 8 are given in this Correction, and the original article has been updated.

Incorrect Fig. 5.

**Fig. 5:**
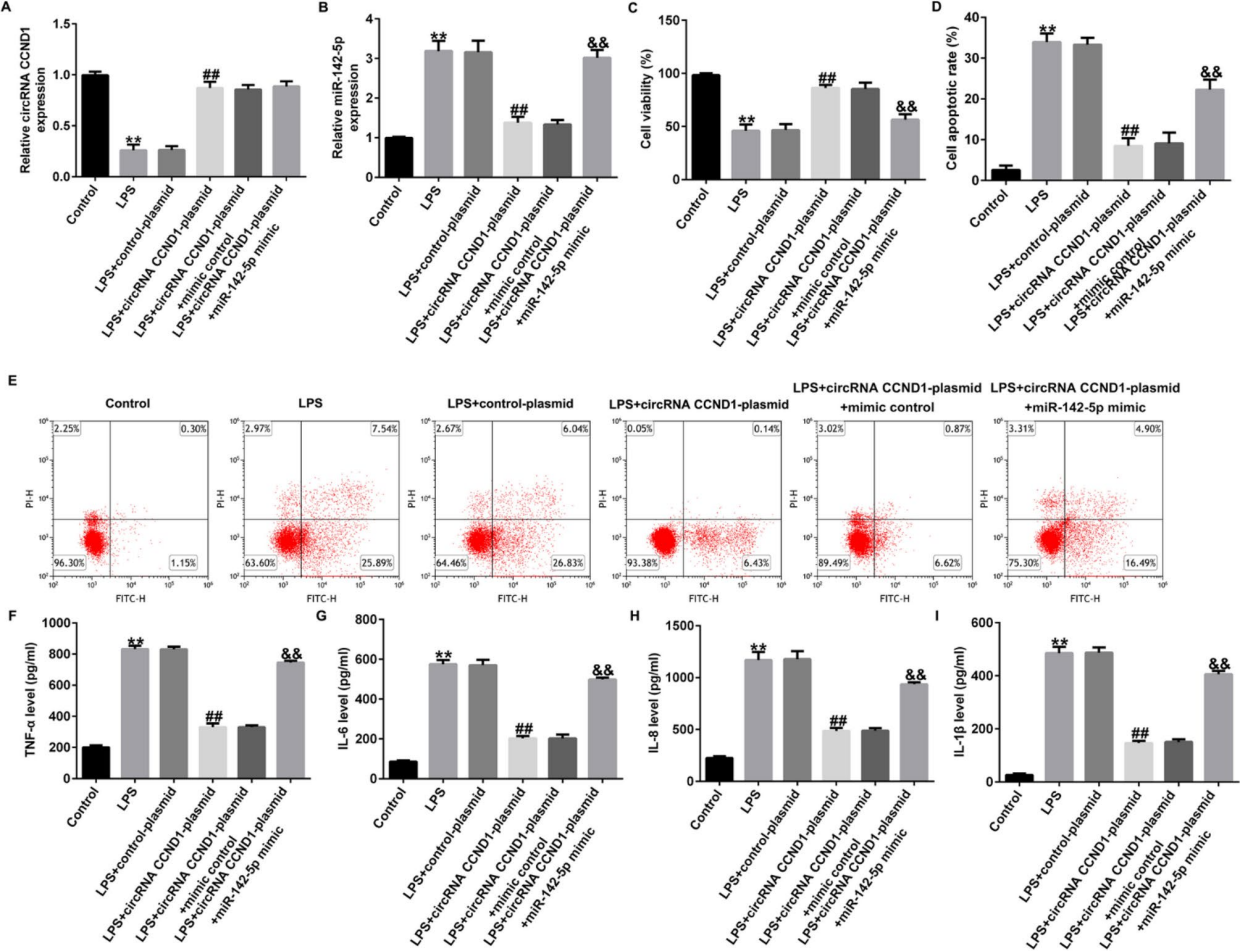
CircRNA CCND1 inhibits LPS-induced Caco-2 cell injury by downregulating miR-142-5p expression. A, B RT-qPCR uncovered the levels of miRNA-142-5p and circRNA CCND1 in different cells. C Cell viability was determined by CCK-8 assays. D, E Apoptosis ratio of LPS-induced cells was detected by flow cytometry. F–I Inflammatory cytokine levels were measured by ELISA. Data are shown as means ± SD of three replicate experiments. **p < 0.01 versus Control; ##p < 0.01 versus LPS + control-plasmid; &&p < 0.01 versus LPS + circRNA CCND1-plasmid + mimic control

Correct Fig. 5.

**Fig. 5:**
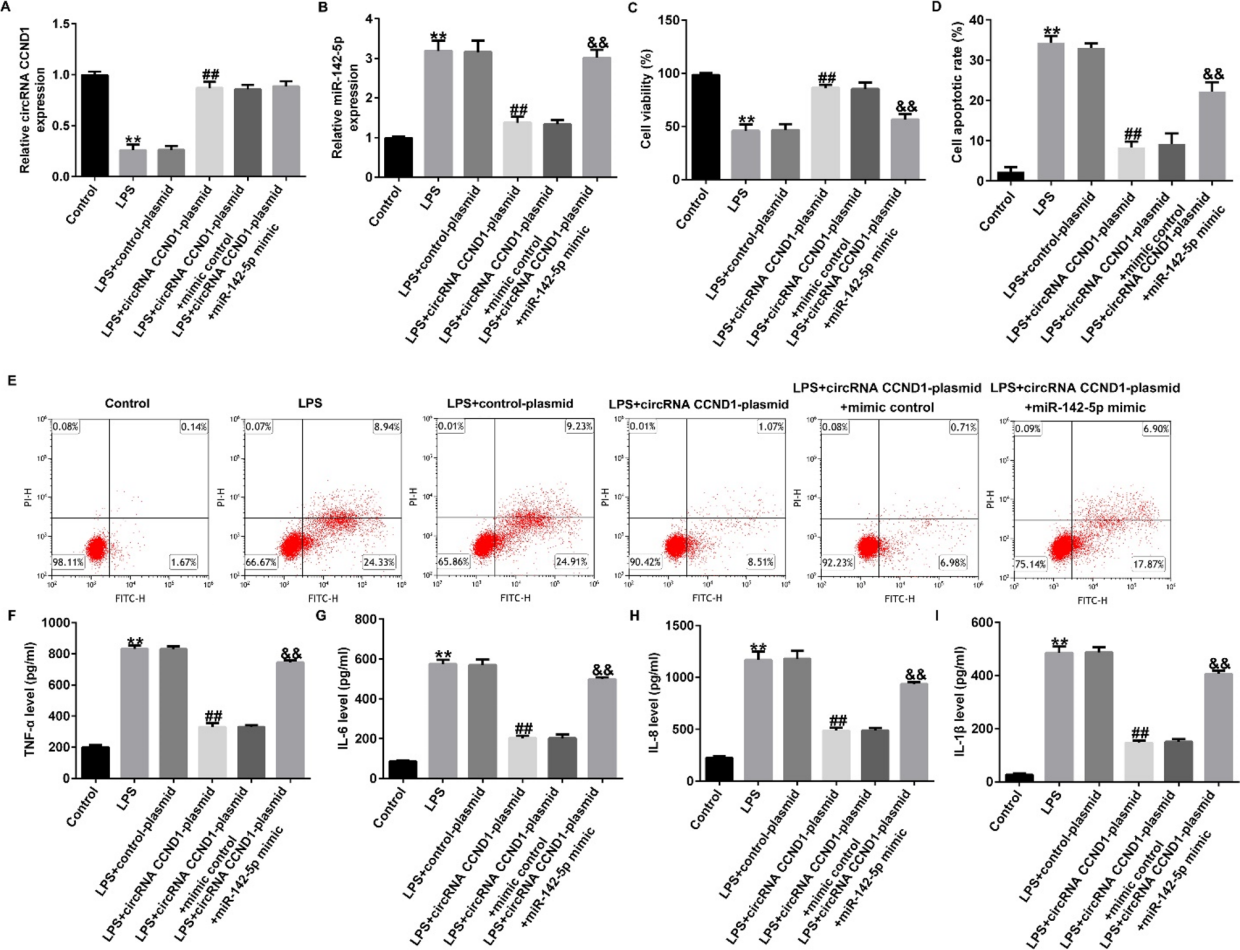
CircRNA CCND1 inhibits LPS-induced Caco-2 cell injury by downregulating miR-142-5p expression. A, B RT-qPCR uncovered the levels of miRNA-142-5p and circRNA CCND1 in different cells. C Cell viability was determined by CCK-8 assays. D, E Apoptosis ratio of LPS-induced cells was detected by flow cytometry. F–I Inflammatory cytokine levels were measured by ELISA. Data are shown as means ± SD of three replicate experiments. **p < 0.01 versus Control; ##p < 0.01 versus LPS + control-plasmid; &&p < 0.01 versus LPS + circRNA CCND1-plasmid + mimic control

Incorrect Fig. 8.

**Fig. 8:**
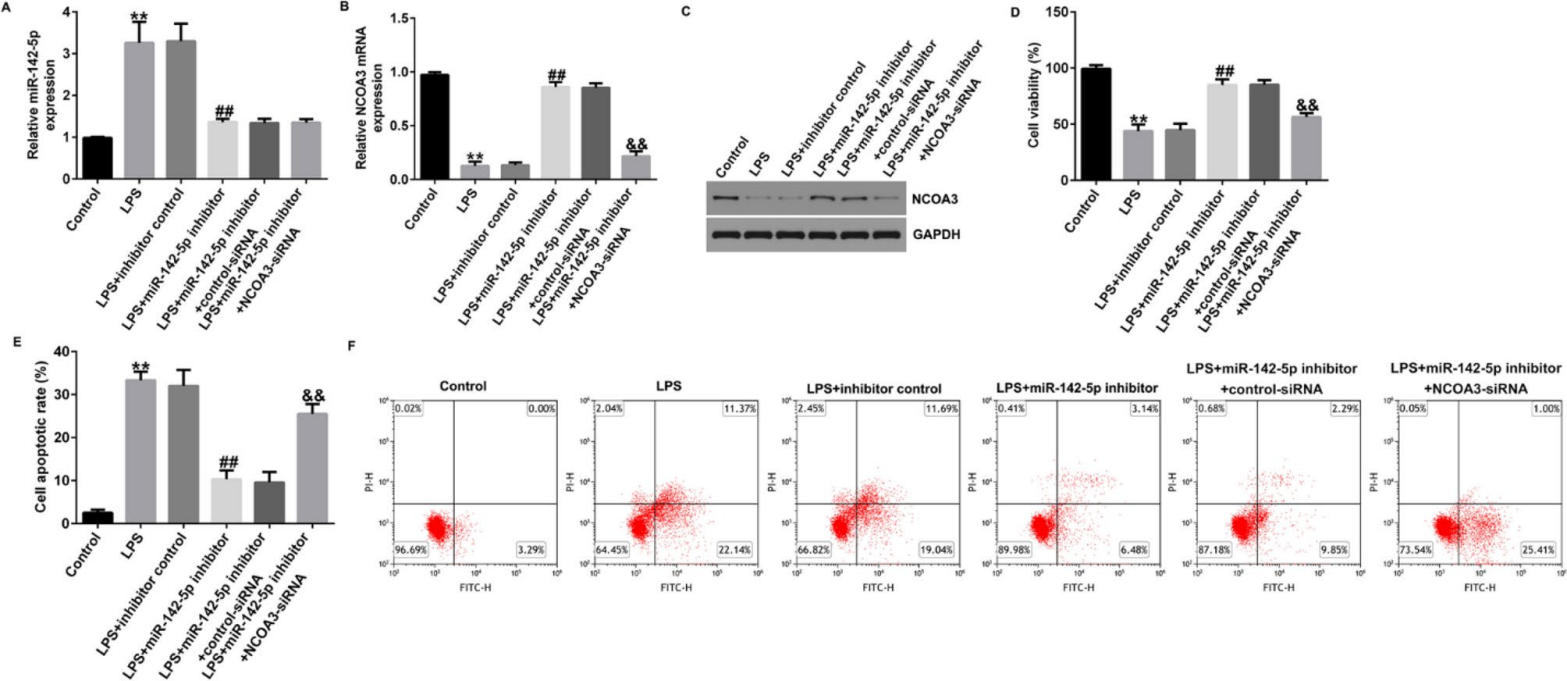
Inhibition of miR-142-5p suppressed LPS-induced cell apoptosis in Caco-2 cells through NCOA3. A, B RT-qPCR revealed the levels of miRNA-142-5p and NCOA3 in different cells. C Western blot assay to analyze NCOA3 protein levels in different cells. D Cell viability was counted using CCK-8 kits. E, F Apoptosis ratio of LPS-induced cells was detected by flow cytometry. Data are shown as means ± SD of three replicate experiments. **p < 0.01 versus Control; ##p < 0.01 versus LPS + inhibitor control; &&p < 0.01 versus LPS + miR-142-5p inhibitor + control-siRNA

Correct Fig. 8.

**Fig. 8:**
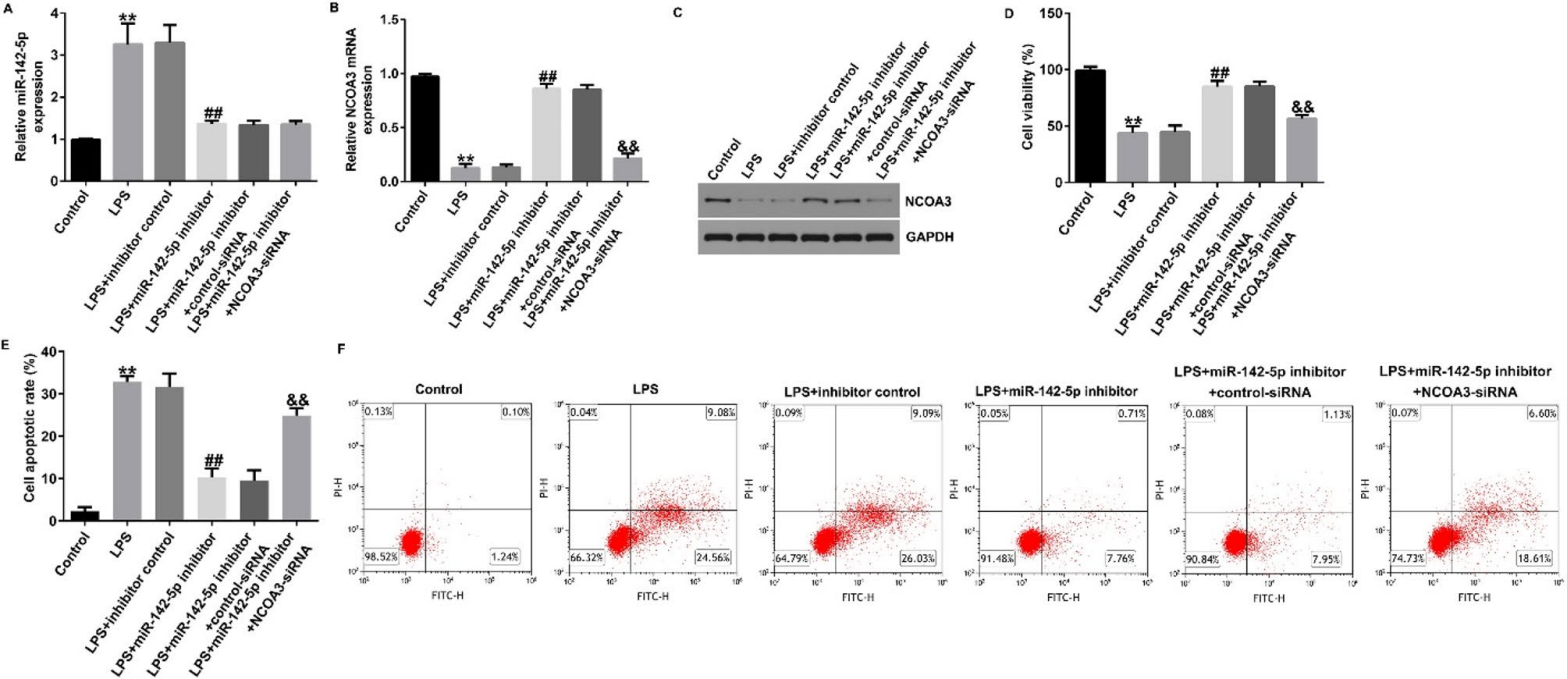
Inhibition of miR-142-5p suppressed LPS-induced cell apoptosis in Caco-2 cells through NCOA3. A, B RT-qPCR revealed the levels of miRNA-142-5p and NCOA3 in different cells. C Western blot assay to analyze NCOA3 protein levels in different cells. D Cell viability was counted using CCK-8 kits. E, F Apoptosis ratio of LPS-induced cells was detected by flow cytometry. Data are shown as means ± SD of three replicate experiments. **p < 0.01 versus Control; ##p < 0.01 versus LPS + inhibitor control; &&p < 0.01 versus LPS + miR-142-5p inhibitor + control-siRNA

