# Supplementary material for: Protective role of circRNA CCND1 in ulcerative colitis via miR-142-5p/NCOA3 axis
Source: BMC Gastroenterol. 2023 Jan 19;23:18. doi: 10.1186/s12876-023-02641-6 (PMC9850594; doi:10.1186/s12876-023-02641-6)
Supplement: Supplementary file 1 — Additional file 1. The original blots. [file 12876_2023_2641_MOESM1_ESM.docx]

**Original blots of Figure 7D**

A: Control; B: inhibitor control; C: miR-142-5p inhibitor; D: miR-142-5p inhibitor+control-siRNA;

E: miR-142-5p inhibitor+NCOA3-siRNA.


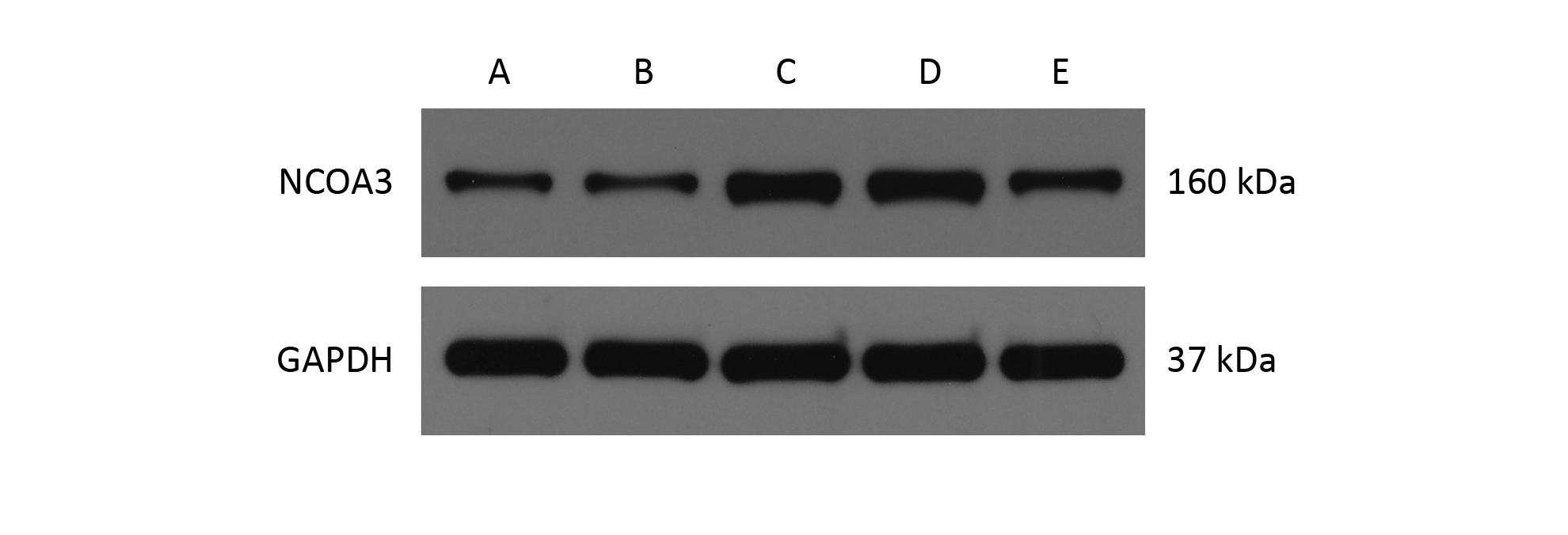


A B C D E


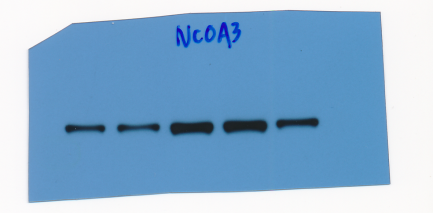


NCOA3: 160 kDa

A B C D E


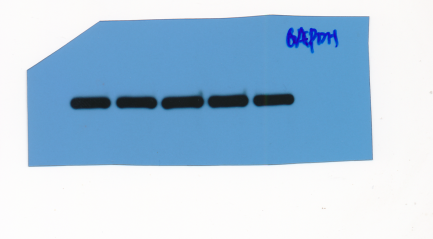


GAPDH: 37 kDa

**Original blots of Figure 8C**

A: Control; F: LPS; G: LPS+inhibitor control; H: LPS+miR-142-5p inhibitor; I: LPS+miR-142-5p inhibitor+control-siRNA; J: LPS+miR-142-5p inhibitor+NCOA3-siRNA


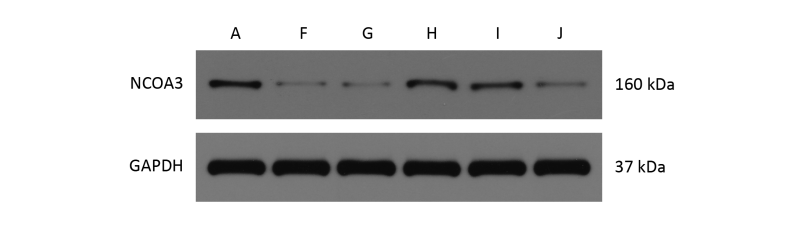


A F G H I J


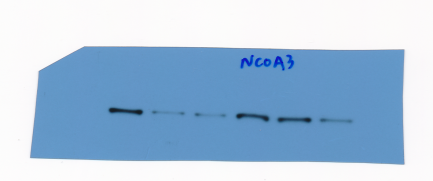


NCOA3: 160 kDa

A F G H I J


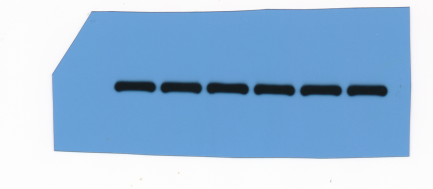


GAPDH: 37 kDa
